# Meta-analysis on studies with heterogeneous and partially observed covariates

**DOI:** 10.11124/JBIES-23-00078

**Published:** 2024-03-01

**Authors:** Tugba Akkaya Hocagil, Hon Hwang, Joseph L. Jacobson, Sandra W. Jacobson, Louise M. Ryan

**Affiliations:** 1 Department of Statistics and Actuarial Science, University of Waterloo, Ontario, Canada; 2 Department of Biostatistics, Ankara University School of Medicine, Ankara, Turkiye; 3 School of Mathematical and Physical Sciences, University of Technology Sydney, Ultimo, NSW, Australia; 4 Department of Psychiatry and Behavioral Neurosciences, Wayne State University School of Medicine, Detroit, MI, USA; 5 Department of Biostatistics, Harvard T.H. Chan School of Public Health, Boston, MA, USA

**Keywords:** cognition, heterogeneous covariates, individual participant data, meta-analysis, propensity score

## Abstract

Individual participant data meta-analysis is a commonly used alternative to the traditional aggregate data meta-analysis. It is popular because it avoids relying on published results and enables direct adjustment for relevant covariates. However, a practical challenge is that the studies being combined often vary in terms of the potential confounders that were measured. Furthermore, it will inevitably be the case that some individuals have missing values for some of those covariates. In this paper, we demonstrate how these challenges can be resolved using a propensity score approach, combined with multiple imputation, as a strategy to adjust for covariates in the context of individual participant data meta-analysis. To illustrate, we analyze data from the Bill and Melinda Gates Foundation-funded Healthy Birth, Growth, and Development Knowledge Integration project to investigate the relationship between physical growth rate in the first year of life and cognition measured later during childhood. We found that the overall effect of average growth velocity on cognitive outcome is slightly, but significantly, positive with an estimated effect size of 0.36 (95% CI 0.18, 0.55).

## Introduction

Meta-analysis is a commonly used approach to combine information across multiple studies to generate a global exposure or treatment effect.^1^ Traditionally, such analyses are derived from summary statistics obtained from published studies. Although this approach is easy to implement, it may be prone to ecological and confounding bias. Individual patient data (IPD) meta-analysis is a popular alternative and has been used in many fields of research,^2^ ranging from public health^3^ to agriculture trials.^4^ Despite its common use, one challenge in IPD meta-analysis, especially in the context of observational data, is that the various studies being combined are unlikely to have information on the same sets of covariates. This problem has been discussed extensively, with authors using the term *systematic missingness* to describe the setting where a particular variable was not collected in a particular study. One potential solution to this problem is to include only covariates that are common to all studies. However, this approach may lead to substantial distortion, as important covariates may be ignored as a result.

Several authors have suggested the use of multiple imputation techniques to handle these kinds of missing covariates,^5–8^ formulating the problem in terms of multilevel data with study viewed as a clustering variable. However, Audigier et al.^8^ compared the most relevant multiple imputation methods for multilevel datasets with systematically missing data and showed that the approach works well only for datasets that include many clusters (studies). There is another issue that makes such approaches cumbersome and impractical. In practice, even when the various studies report on essentially the same covariates, there will often be differences in precisely how variables are defined or quantified. For example, while all the studies might include covariates related to socioeconomic status, it is likely that they will vary in the specifics of how this variable (ie, socioeconomic status) is quantified. Psaki et al.^9^ raise this issue in the context of multi-country birth cohort studies. They discuss how multi-country studies pose a challenge to measuring socioeconomic status because variables used to measure socioeconomic status may have different meanings across populations. They sought to provide guidance on measuring socioeconomic status accurately in epidemiological studies of diverse populations to address this challenge.^9^ Another example is that a variable such as maternal age may be grouped and categorized different from one study to another. A more practical strategy is needed to facilitate the conduct of meta-analysis in such settings.

In this paper, we argue that a propensity score approach is simple, practical, and can work well in meta-analysis settings. One of the key underlying assumptions of a valid propensity score analysis is that all variables that affect treatment assignment and outcome have been measured. The propensity score method was first introduced by Rosenbaum and Rubin^10^ as a way of addressing the lack of randomization in observational studies. The idea is that the propensity score acts as a balancing factor, adjusting for the fact that the distribution of measured baseline covariates may differ between treated and untreated subjects, and implying that among subjects with the same propensity for exposure, treatment is conditionally independent of the covariates. Consequently, this balancing property suggests that estimates of the exposure effect, uncontaminated by any of the measured covariates, can be obtained by estimating the effect of exposure within groups of people with the same propensity score. Within such a group, any difference in outcome between the exposed and unexposed subjects is not attributable to the measured confounders.^11^

Rubin and Thomas^12^ suggest incorporating into a propensity score model all variables believed to have a relationship with the outcome, regardless of their association with exposure. Therefore, it is important to consider the implications of this assumption in our context, namely, where multiple studies are being combined in a meta-analysis. As discussed previously, it is likely that the various studies will differ in terms of the precise nature of the covariates being measured. Before utilizing the methods described in our paper for combining estimates from a covariate-adjusted meta-analysis, it is critical to evaluate whether each study has measured enough relevant covariates to provide adequate control for confounding so that the treatment effect estimates from each study are unbiased, on average. Later in the paper, we will demonstrate through a small simulation that if one or more of the studies being analyzed lack adequate covariate adjustment, the estimates derived from those studies will exhibit bias, thereby introducing some bias in the overall meta-analysis.

When Rosenbaum and Rubin^10^ first introduced the propensity score method, it was in the context of estimating the causal effect of a binary treatment variable. They estimated the propensity score as the conditional probability of being treated given the subject’s covariates.^13,14^ Rosenbaum and Rubin showed that if the baseline covariates of the subjects are sufficient to control for confounding, then adjusting for the propensity score is also sufficient.^14^ Imai and van Dyk^15^ extended this framework to accommodate a continuous exposure variable and referred to the resulting propensity score as a generalized propensity score. The generalized propensity score approach has been used in settings involving continuous exposure variables related to labor earnings,^15,16^ medical expenditures,^15^ and birth weight,^17^ among others.

In this work, we propose an approach based on the generalized propensity score as a strategy to adjust for potential confounders that vary by study in the context of IPD meta-analysis. Although Imai and van Dyk^15^ suggested several ways to use the generalized propensity score, we use the approach that involves simply including the estimated propensity score as a covariate in addition to the main exposure or treatment effect in a regression model adjustment^18^ via the estimated propensity score summarizes all the characteristics of the individual subjects into a single covariate,^18^ then can be used as a single additional covariate in an outcome regression model. Indeed, this strategy resolves the challenge associated with having different covariates in each study by having the estimated propensity score as the single additional covariate in each study’s outcome model. Consequently, we are back in the setting where the same regression models are being fit across all the studies. Without this strategy, especially for conducting the IPD meta-analysis, one has to either include covariates that are common to all studies or use multiple imputation techniques to impute systematically missing covariates. Of course, the critical caveat applies that the studies being combined have all, individually, measured adequate covariates to adjust appropriately for confounding. We will demonstrate appropriate usage of the estimated propensity score as an additional covariate in both one-stage and two-stage IPD meta-analyses.

In our motivating example, we encounter a problem common to almost all applied settings, namely, that there are missing values for some of the covariates needed for computing the propensity score within each study. Multiple imputation is a commonly used approach for handling missing data on covariates, although relatively little is known about its use in the context of propensity score estimation and IPD meta-analysis. Specifically, combining multiple imputation with the propensity score methodology and meta-analysis raises an important question about when to apply Rubin’s rule. We demonstrate the steps we have taken to impute missing data via multiple imputation, adjust for potential confounders using the propensity score method, and, finally, synthesize information across studies via one-stage and two-stage IPD meta-analysis.

In next section, we review and summarize the methods used to estimate the propensity score in the presence of missing covariates and demonstrate the use of the estimated propensity score in IPD meta-analysis. We then illustrate the methods using child growth data from the Healthy Birth Growth and Development Knowledge Integration (HBGDki) project, sponsored by the Bill and Melinda Gates Foundation. Further information about the project can be found at https://www.kiglobalhealth.org/. We then discuss the result of the analysis in the application section, followed by a discussion of the limitations and the strengths of the proposed approach.

### Multiple imputation, propensity score methodology, and meta-analysis

In this section, we describe the use of propensity score adjustment as a tool for combining data from multiple cohorts that vary according to the confounders that have been measured. To begin with, we assume that there are no missing data but also discuss how the strategy can be adapted to handle missing data using multiple imputation. Two different approaches to IPD meta-analysis are considered, one based on a two-stage strategy and the other based on mixed-effects modeling. We start with a brief review of the use of propensity scores for confounder adjustment.

### Propensity score estimation

Consider the setting where we wish to model the causal effect of a continuous treatment or exposure variable Z on a continuous outcome Y in the presence of a set of p potential confounding variables, which we denote $X_{1}$, $X_{2}$ to $X_{p}$. For ease of discussion from here on, we will simply refer to Z as an exposure variable. To facilitate a brief review of how propensity score analysis works, we consider for now only the context of a single study. To address the effect of confounding, Imai and van Dyk suggested regressing the exposure variable Z on the set of observed covariates, using ordinary least squares regression. This propensity score model^15,19^ can be written as follows:

(1) $$Z_{j}=\alpha_{0}+\alpha_{1}X_{1j}+\alpha_{2}X_{2j}+\ldots +\alpha_{p}X_{pj}+w_{j}$$

in which $Z_{j}$ is the exposure variable measured on subject j and $X_{1\text{j}}$, $X_{2\text{j}}$, …, $X_{\text{pj}}$ are the potential confounding variables or covariates measured on that same subject. In the regression model (1), $w_{\text{j}}$ is an error term assumed to have mean 0.

Because we are focusing on only a single study, we do not include any extra subscript, k, to indicate study, although we will do so later. The estimated propensity score can be obtained for individual j as the predicted value of $Z_{j}$, given all the covariates, obtained after fitting the model. We denote this propensity score by $S_{j}$:

(2) $$S_{j}=Pred\left(Z_{j}|X_{1j},X_{2j},\ldots ,X_{pj}\right)={\hat{\alpha }}_{0}+{\hat{\alpha }}_{1}X_{1j}+{\hat{\alpha }}_{2}X_{2j}+\ldots +{\hat{\alpha }}_{p}X_{pj}$$

and where the ${\hat{\alpha }}_{\text{s}}$ are the estimated regression coefficients obtained by fitting equation (1) via least square using standard regression analysis software (eg, we used package lm in R statistical software [R Foundation for Statistical Computing, Vienna, Austria]). In the application that motivates our work, for example, the exposure variable Z is the growth velocity, defined as the rate of change in HAZ (height for age z score) over the first year of life, and we defined the propensity score as the conditional density function of the growth velocity given all the covariates including birth weight, sex, mother’s race, mother’s education, maternal age at birth, number of still births, and parity. We will return to the application later.

The seminal work by Rosenbaum and Rubin^10^ showed that once the propensity score has been estimated, the effects of confounding can be eliminated through several different means, including propensity score matching, stratification according to the propensity score, and inverse probability weighting using the propensity score or using the propensity score directly as a covariate. For this paper, we focus on the latter strategy because it lends itself particularly well to our context. More precisely, we will consider linear regression models that predict the outcome of interest as a function of the exposure variable Z and the propensity score S:

(3) $$Y_{j}=\delta_{0}+\delta_{1}Z_{j}+\delta_{2}S_{j}+e_{j},$$

where $Y_{\text{j}}$ is the outcome variable observed on individual j, $Z_{j}$ is the corresponding treatment variable, and $S_{j}$ is the estimated propensity score for that subject. The error term $e_{j}$ is assumed to have mean 0.

An advantage of using the estimated propensity score in covariate adjustment compared with traditional multivariable regression is that because the propensity score is scalar, it allows us to use a simpler model for confounding adjustment that might not be possible with high-dimensional confounders.^20^

### Meta-analysis

In this subsection, we consider a scenario involving K different studies, each with a common exposure variable, but with potentially different sets of confounders. While adjustment via traditional multivariable regression would result in a different outcome model for each study, use of propensity score adjustment results in a common model (3) across all the studies. Because the propensity score calculated for each study may be based on slightly different sets of covariates for each study, it is important to allow the effect of the propensity score on the outcome to vary by study. The following section provides some more detail about how this can be done. We describe 2 possible IPD meta-analysis approaches to estimate the average exposure treatment effect from multiple independent studies, assuming for now that all the covariates needed to compute the propensity scores are fully observed and that a propensity score has been estimated separately for each study, using the strategy described in the previous section.

### Two-stage individual participant data meta-analysis

In a two-stage IPD meta-analysis, we start by fitting separate linear models for each of the K separate studies. It is useful to now extend our previous notation to include two subscripts, with j indicating individual as before, but now with an additional k to indicate study. For study k, we fit the following regression model:

(4) $$Y_{jk}=\delta_{0k}+\delta_{1k}Z_{jk}+\delta_{2k}S_{jk}+e_{jk},$$

where $\delta_{1k}$ represents the effect of a 1-unit increase in the exposure or treatment variable $Z_{\mathrm{jk}}$ on the mean outcome in study k, given the propensity score $S_{\mathrm{jk}}$. The parameter $\delta_{2k}$ characterizes the effect of the propensity score for a given level of alcohol exposure in study k, and $e_{\mathrm{jk}}$ is a zero mean error term. If desired, non-linear effects of the propensity can easily be accommodated using gam models.^21^ In the second stage, we combine the estimates of the K different study-specific treatment effects ${\hat{\delta }}_{11},{\hat{\delta }}_{12},\ldots ,{\hat{\delta }}_{1k}$ to obtain an overall estimate. To account for study-to-study heterogeneity, we employ a random-effects meta-analysis model, where both the study-specific estimates and the overall result are realizations from statistical distributions.^22^ Specifically, we assume ${\hat{\delta }}_{1k}=\delta_{1k}+e_{k}$, where $\delta_{1k}$ represents the true treatment or exposure effect in study k and $e_{k}\sim N\left(0,{se}_{k}^{2}\right)$ where ${\mathrm{se}}_{k}$ is the estimated standard error of ${\hat{\delta }}_{1k}$, obtained at the first stage analysis for the kth study. The true study-specific effects are assumed to vary across study according to a normal distribution. Specifically, we assume ${\text{δ}}_{1\text{k}}\sim \text{N}(\text{θ},{\text{τ}}^{2})$ so that θ can be interpreted as the “global effect” of a 1-unit increase in the treatment across all studies and ${\text{τ}}^{2}$ reflects the extent of heterogeneity of the study-specific exposure effects. When there are a very large number of studies being combined, it may be possible to include study-specific covariates in the specified model for ${\text{δ}}_{1\text{k}}$, but in most cases, the simple model will suffice and θ will be t the parameter of interest.^23^ It is straightforward to fit this model by noting that ${\hat{\delta }}_{1k}∼N\left(\theta ,\left(se^{2}+\tau^{2}\right)\right)$, then using optimization software to estimate θ and ${\text{τ}}^{2}$ via maximum likelihood. We used the package *optim* in R.^24^ Conducting a meta-analysis using random-effects modeling to capture study-to-study variability is standard practice.^25^

### One-stage individual participant data meta-analysis

When we have all the individual-level data across all studies, an alternative meta-analysis method is to fit a linear mixed-effects model using all the data in one step. To see this, it is helpful to re-express model (4) and associated assumptions on ${\text{δ}}_{1k}$ as follows:

(5) $$Y_{jk}=\delta_{0}+\left(\theta +u_{k}\right)Z_{jk}+\delta_{2k}S_{jk}+e_{jk},$$

where $u_{k}$ is a normally distributed random effect, specifically $u_{k}\sim \text{N}(0,{\text{τ}}^{2})$, and $e_{\mathrm{jk}}\sim \text{N}(0,{\text{σ}}^{2})$. The term $\delta_{2k}$ corresponds to the coefficient of the propensity score in the kth study. This is treated as a fixed rather than a random effect. If we think of writing out the data in long form, there would be a variable Y corresponding to outcome, Z corresponding to treatment or exposure, S corresponding to propensity score, and an additional variable, say this is denoted *STUDY*, indicating the study to which an observation corresponds. In specifying the linear mixed model, the effect of Z would be modeled as random across *STUDY,* and there would be an interaction between S and *STUDY*. Technically, the error variance ${\text{σ}}^{2}$ should be allowed to vary by study, although in practice it will often be adequate to assume a common value. For more discussion of mixed-effects modeling, see the book by Fitzmaurice, Laird, and Ware.^26^ The detailed SAS and R code to fit this model is provided in the supplemental content 1, http://links.lww.com/SRX/A38.

### Multiple imputation

In practice, it is inevitable that some of the variables needed to compute the study-specific propensity scores will have missing values. One can perform a complete case analysis by ignoring subjects who have missing data for any of the relevant covariates. However, the literature on missing data is clear regarding the disadvantages of failing to address missing data using more principled solutions. Removing subjects with missing data may cause bias and lead to increased uncertainty, and it is generally recognized as inefficient use of data. Many suggestions have been made on how to handle missing data, and these suggestions can be roughly classified into weighting- and matching-based methods, likelihood-based methods, and multiple imputation. Among these, multiple imputation has proven to be an efficient method that is easy to implement in a wide range of missing data problems. It is the approach we explore here, and it works particularly well in our context.

In general, there are 2 approaches to imputing multivariate data, either via joint modeling or through a fully conditional specification.^27^ In settings where we have different types of variables (eg, a mix of continuous and discrete), joint modeling can be quite complex and satisfactory solutions are not readily available. The fully conditional specification offers more flexibility to handle missing data in this setting, as a specific imputation model is specified for each partially observed variable. One of the most commonly used implementations of the fully conditional specification method is the multivariate imputation by chained equations (MICE).^28^ Due to its flexibility and software availability, we employ the R package MICE to handle missing data on confounders in our work. For simplicity and because we will be discussing this further in the next section, we go back to considering data from a single study (so for ease of exposition, the study-specific subscript k will again be omitted for now). Our analysis goal can be thought of as fitting a set of paired equations, the first being the regression model (1) predicting the exposure variable as a function of covariates and the second a simple regression model predicting the outcome of interest as a function of the exposure variable and propensity score, computed from the first regression model. More precisely, we need to fit the following pair of equations:

(6) $$Z_{j}=\alpha_{0}+\alpha_{1}X_{1j}+\alpha_{2}X_{2j}+\ldots +\alpha_{p}X_{pj}+w_{j},$$

(7) $$Y_{j}=\delta_{0}+\delta_{1}Z_{j}+\delta_{2}S_{j}+e_{j},$$

where, as discussed earlier, $Z_{j}$ is the exposure variable of interest for subject j; $X_{1j}$, $X_{2j}$, …, $X_{pj}$ are the corresponding p covariates measured on that subject; $Y_{j}$ is the outcome variable; $S_{j}$ is the estimated propensity score based on the first equation; and, finally, $w_{j}$ and $e_{j}$ are error terms. The principle of multiple imputation is to generate a set of plausible values for the missing variables by generating values based on the predictive distribution of these variables given the observed data. The MICE algorithm initializes with a simple random draw from the data for any missing variables, sets up a series of regression models predicting each variable as a function of all the others, and then iterates until convergence. The coupled nature of equations (6) and (7) make it clear that in conducting the imputation, values of Y also need to be included, although technically the missing values affect only computation of the propensity scores based on (8). The resulting predictive models can be used to generate M complete data sets, which can then be analyzed independently to produce M different estimates of each parameter. For example, consider estimation of $\alpha_{1}$, the coefficient associated with the first covariate, ${\text{X}}_{1}$. The M estimates from the M imputed datasets would be ${\hat{\alpha }}_{11}$, ${\hat{\alpha }}_{12}$, ${\hat{\alpha }}_{13}$, …, ${\hat{\alpha }}_{1M}$, with associated estimated standard errors, $\hat{se}({\hat{\alpha }}_{11})$, $\hat{se}({\hat{\alpha }}_{12})$, $\hat{se}({\hat{\alpha }}_{13})$,……… $\hat{se}({\hat{\alpha }}_{1M})$, so that the pooled estimate would then be

(8) $${\hat{\alpha }}_{1pooled}=\frac{1}{m}\sum_{m=1}^{M}{\hat{\alpha }}_{1m},$$

and the pooled standard error would be

(9) $$\hat{se}\left({\hat{\alpha }}_{1pooled}\right)=\sqrt{W\left(1+\frac{1}{M}\right)B},$$

where W averages the squared estimated standard errors computed within each of imputed datasets,

(10) $$W=\frac{1}{M}\sum_{m=1}^{M}{\left(\hat{se}\left({\hat{\alpha }}_{1m}\right)\right)}^{2},$$

and B measures the variability in the estimates themselves across the imputed datasets:

(11) $$B=\frac{1}{M-1}\sum_{m=1}^{M}{\left({\hat{\alpha }}_{1m}-{\hat{\alpha }}_{1pooled}\right)}^{2}.$$

Inference can now proceed as usual, using ${\hat{\alpha }}_{1pooled}$ and ${\hat{se(}\hat{\alpha }}_{1pooled})$.

### Simulation studies

We assessed the performance of our proposed method through a small simulation study. For this experiment, we generated a dataset consisting of 10 studies, each with 200 participants. For each study, we generated 3 covariates ($X_{1}$, $X_{2}$, $X_{3}$), an exposure variable (E), and an outcome variable (Y). Details about assumed parameter values (Table S1), underlying data generation mechanisms, and our results are provided in the supplemental material, Supplemental Digital Content 1, http://links.lww.com/SRX/A38. Briefly, the 3 covariates were generated to ensure that they were each genuine confounders, but also that they had study-to-study heterogeneity, reflecting the key concept that the studies being combined measure similar, although not identical, covariates.

We assessed the performance of our proposed method of conducting meta-analysis of studies with heterogeneous covariates on a range of metrics including empirical bias, average model-based standard error, empirical standard error, and coverage probability^29^ (Table S2). We found that both the one-stage and the two-stage meta-analysis methods did equally well in terms of empirical bias. Coverage probabilities were good for both methods, although they were closer to the desired level for the two-stage method. This is most likely because the one-stage method did not allow for study-to-study heterogeneity in error variances.

Crucially, we used the simulations to assess how the methods work in settings where one or more of the studies being combined have not measured sufficient covariates to properly adjust for confounding. We considered 2 simulation scenarios (A and B). In scenario A, we assume that all the relevant covariates ($X_{1}$, $X_{2}$, $X_{3}$) are completely observed. Under this scenario, we estimated the propensity score for each study using all 3 covariates, as described in previous sections. In scenario B, we assume data on two covariates ($X_{2}$ and $X_{3}$) were not collected for Study 2 and Study 6, respectively, although covariate $X_{1}$ was completely observed across all the studies. Under scenario B, we estimated the propensity score in two ways. Following our proposed strategy, we estimated the propensity score separately for each study using all available data. This means that the propensity score for Study 2 was estimated using only $X_{1}$ and $X_{3}$, while the propensity score for Study 6 was estimated using covariates $X_{1}$ and $X_{2}$. For comparison purposes, we also considered a more conventional approach of including only covariates that are common to all studies (in this setting, this means only using $X_{1}$ and ignoring ${\text{X}}_{2}$ and ${\text{X}}_{3}$, even when available).

**Figure 1:**
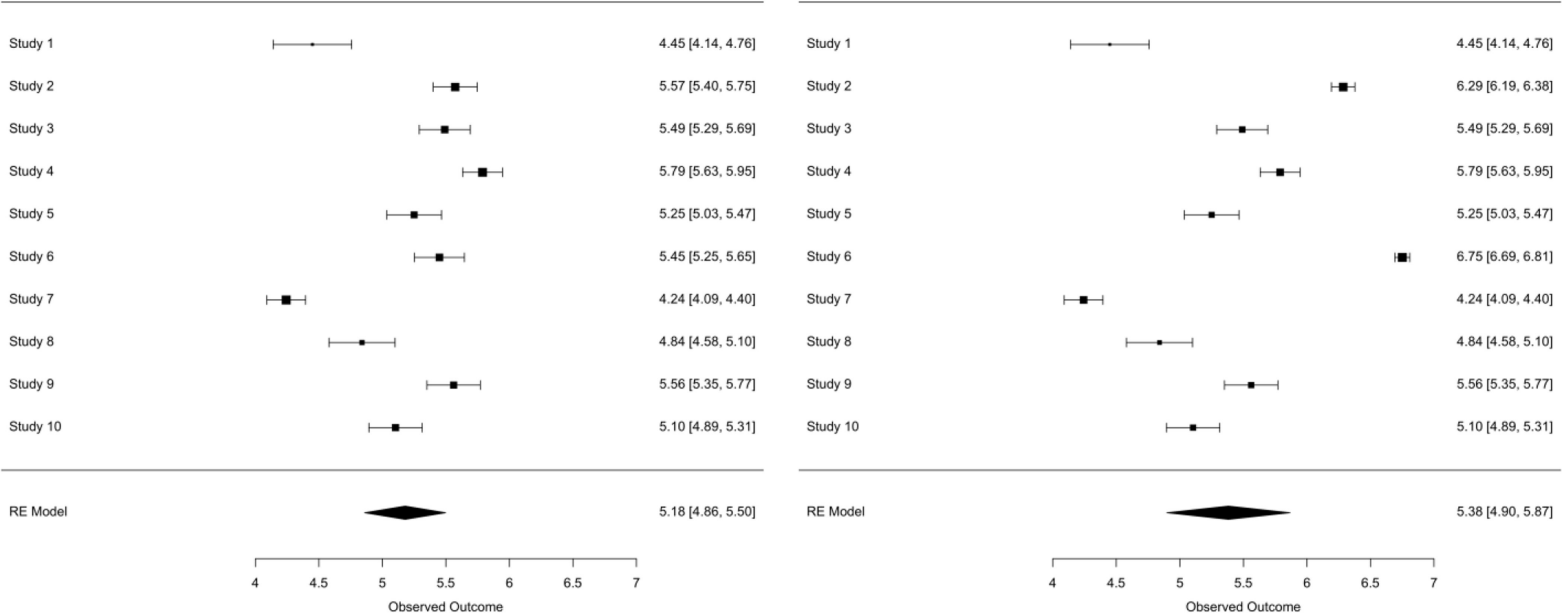
Forest plots from the two random-effects meta-analyses conducted for simulation scenarios A *(left panel)* and B *(right panel)*.

As expected, analysis under scenario B results in biased estimation of the overall exposure effect and coverage probabilities are too low. For the case where all available covariates are used, the bias is relatively small. However, the bias is substantial when analysis was based on the one covariate that was fully observed across all the studies. Figure 1 represents a comparison of forest plots that are obtained from the two-stage meta-analysis conducted under a single simulation, with scenario A in the left panel and scenario B on the right. The left-hand panel illustrates the classic pattern observed in meta-analysis, namely, all the study-specific estimates varying around a common value (in this case the “true” value was 5) and the overall estimate being close to the true value, with standard errors reflecting both sampling variability and study-to-study heterogeneity. In the right-hand panel, it is immediately apparent that studies 2 and 6 look different from the others, with both have exposure effect estimates biased toward the null. The overall estimate shows a slight bias toward the null, although with increased standard error reflecting an apparent increase in study-to-study variability.

### Application to child growth data

We use data from the HBGDki initiative to illustrate the use of propensity scores and multiple imputation to synthesize information across studies with heterogeneous and partially missing covariates. Although the project included data on several hundred different studies, only 9 had usable data related to growth rates in the first year of life as well as cognition measured in childhood. Table 1 summarizes the available data. Among the included studies was the very large Collaborative Perinatal Project,^30^ which had 11 different sites, that we treat as separate studies in our analysis (those starting with acronym cpp in Appendix I). Other studies (and the acronyms used in the table) were as follows: Consortium of Health-Orientated Research in Transitioning Societies (COHORTS)-Phillipines^31^ (cph), CMC Vellore Birth Cohort 2002^32^ (cvb), JiVitA-3: Impact of antenatal multiple micronutrient supplementation on infant mortality^33^ (jta), Promotion of Breast Feeding Interventional Trial^34^ (pbt), Peru Persistent Diarrhea study^35^ (pvd), Social Medical Survey of Children attending Child health Clinics^36^ (scc), MRC Keneba^37^ (nhb), and Growing Up in Singapore Towards healthy Outcomes^38^ (gto).

Table 1 also summarizes sample sizes per study (or site within the study for the CPP), which ranged from 147 to 8676. All studies had repeated growth data available, starting from birth and continuing throughout childhood. Child cognition was assessed using Bayley Scales of Infant and Toddler Development as early as 1–2 years of age in several studies, as well as with standardized tests for general cognition and IQ at school age.

Our goal was to use meta-analysis to assess the relationship between physical growth rate in the first year of life with cognition measured later during childhood. One challenge was that the studies varied with respect to the available covariates, the timing of various growth measures, and the precise nature of the measured outcomes. Some children were missing some of the covariate measures as well. We discuss these various aspects first before describing the use of multiple imputation and propensity score methods to facilitate a meta-analysis. Table 1 summarizes the information related to the type of cognitive function measures, the number of subjects, the timing of measures of cognition during childhood, and the type of covariates collected by each study. While most of the studies collected information on gravidity, parity, sex, and socioeconomic status, some of the studies provided more information on unhealthy behaviors during pregnancy, such as maternal smoking and alcohol consumption. A complete list of covariates collected in each study is presented in Appendix I.

Child growth is commonly characterized using height or weight for age z scores (HAZ and WAZ, respectively) from the World Health Organization (WHO) standardized growth charts.^39,40^ The WHO standardized growth charts allow a child’s physical growth to be quantified at a particular age, compared with the population distribution of children of the same age and sex.^39^ Children who are found to have low HAZ or WAZ scores at any age are said to be stunted and may receive further follow-up and intervention. In the HBGDki initiative, there was interest in studying how growth may change over time. The term *growth velocity* refers to the rate of change in HAZ and WAZ over a particular time period. As suggested by Anderson et al.,^41^ we obtained growth velocity measures for each child by fitting a broken-stick model, which is a piecewise linear spline model,^26^ with child-specific random effects corresponding to the slopes in each segment. For our purpose, we extracted the child-specific slopes corresponding to the first year of life and considered these as measures of growth velocity. An advantage of this approach is that it does not matter when measurements were taken as long as there are enough measurements within the first year of life to quantify growth during that time period reliably. Our goal was then to examine the relationship between growth velocity in the first year of life with cognition measured later in childhood.

The studies included in our meta-analysis used a range of tests to measure child cognition. For this analysis, we focus on the global cognitive tests, which are well known assessments that can be adapted for use in different countries and cultures. Some examples of these global cognitive tests are Bayley Scales of Infant Development,^42^ the Wechsler Intelligence Scale for Children,^43^ and the Wechsler Abbreviated Scale of Intelligence.^44^ In our analysis, we use the cognitive test results from 2–7 years of age. Scores were standardized to the same scale (mean 100, SD 15) before analysis to ensure comparability of estimated exposure effects.

The methods outlined in the previous sections on multiple imputation, propensity score methodology, and meta-analysis address various challenges when attempting to perform a meta-analysis on the estimated coefficients for the average growth velocity in the first year. As described, the goal is to use the same model across all the studies using propensity scores derived from multiply imputed datasets.

In many studies within HBGDki, these socioeconomic covariates are missing in the sense that values of the covariate were not recorded for some subjects. We could ignore the subjects with missing values and use complete case analysis; however, as there are many socioeconomic covariates, we would have to discard many subjects. Thus, complete case analysis would provide an inefficient model. Moreover, the model would be biased because the data would include only those subjects with all the covariates. To handle missing data properly, we impute the missing values using the MICE package in R. To avoid instability in the estimation process, we did not impute variables for which the frequency of missing data was more than 50% and omitted any such variables from the analysis. We also did not impute categorical variables with multiple levels for which only one level was observed in the dataset.

**Figure 2:**
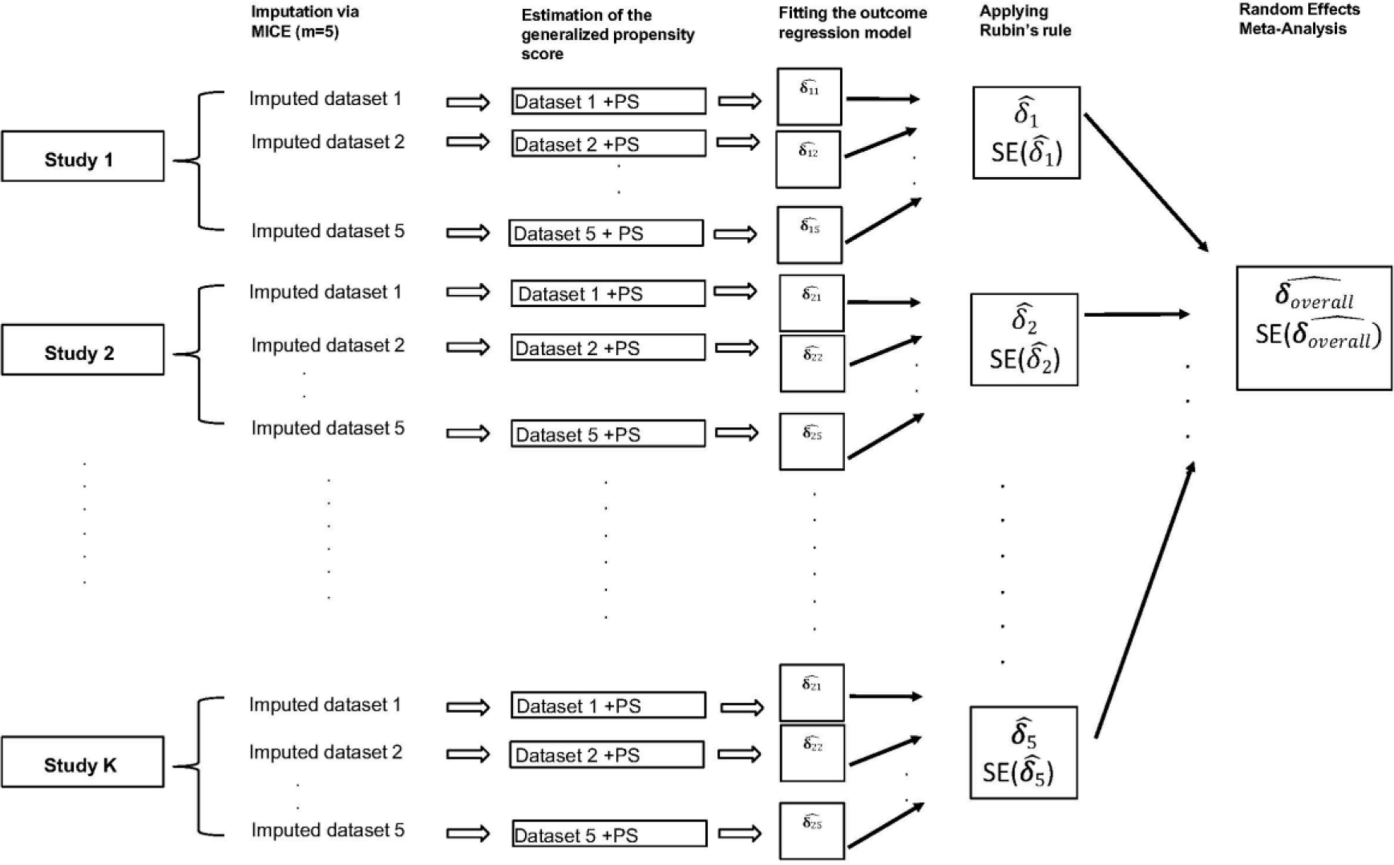
RR then MA approach: the sequence of operation is to first impute using multiple imputation, followed by estimation of the propensity scores, and then fitting the regression model of interest. These estimated regression coefficients {$\delta_{11}$, $\delta_{12}$, $\delta_{13}$} are then pooled. Each study now has a pooled estimate, which we use in our random-effects meta-analysis.

**Figure 3:**
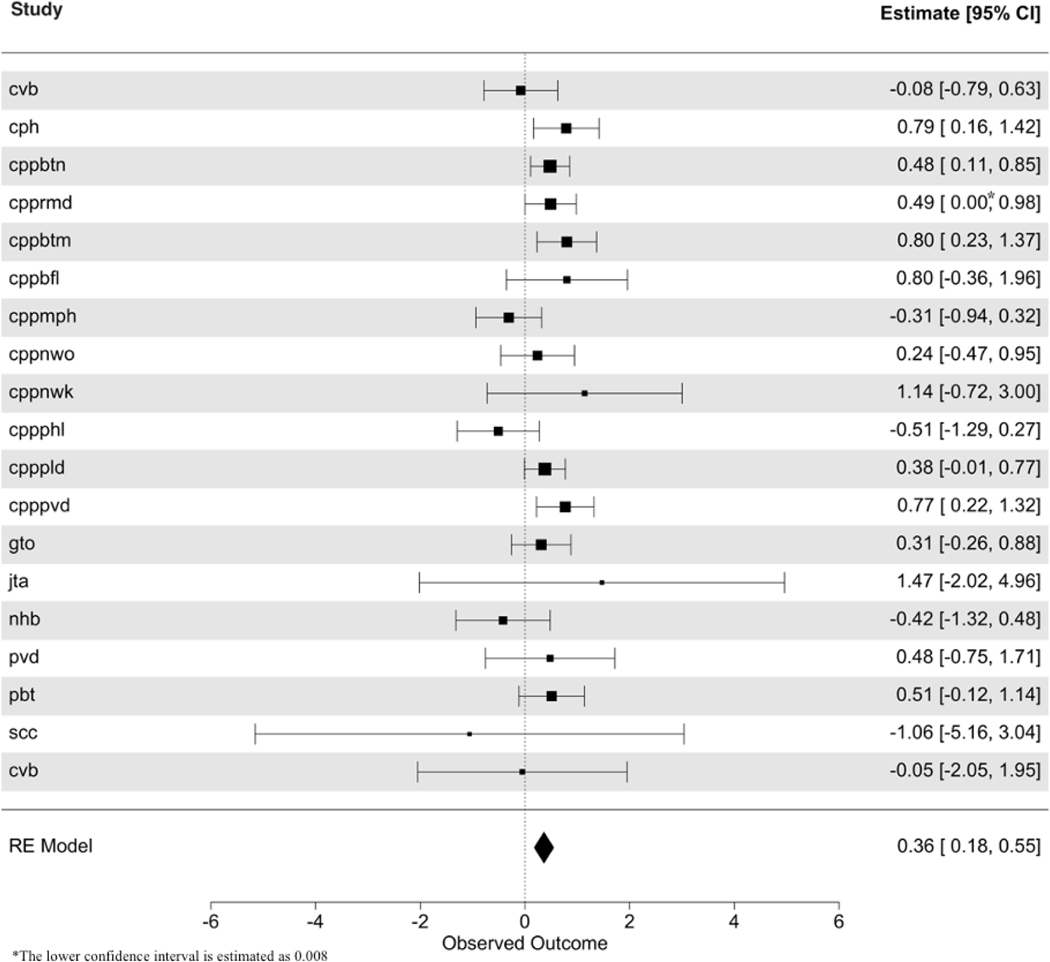
Forest plot from the random-effects meta-analysis of the estimated regression coefficients with covariate adjustment using propensity scores.

In the previous section, we describe the two approaches to analysis workflow to consider multiple imputed datasets generated by MICE, namely “RR then MA,” which involves applying Rubin’s rules first, then performing the meta-analysis, or its alternative “MA then RR.” In this work, we use the “RR then MA” approach for the two-stage IPD meta-analysis, as illustrated in Figure 2. Following the “RR then MA” approach, we first imputed the missing values within each study. The imputation process generates multiple imputed datasets. We then estimated the propensity scores using the propensity score model where our treatment variable is the average first-year growth velocity of the subjects and, as shown in Figure 2, we appended the estimated propensity score as an additional variable in each imputed dataset. Then we fitted a regression model predicting cognition measured later during childhood as a function of physical growth rate in the first year of life and the estimated propensity score to obtain an estimate of the parameter of interest—the effect of physical growth rate in the first year on childhood cognition in each imputed dataset. The estimates from each imputed dataset were then combined, as shown in Figure 2. The forest plot in Figure 3 illustrates that the overall effect of average growth velocity on cognitive outcome is slightly positive, with an estimated effect size of 0.36 and a 95% CI of 0.18, 0.55.

Figure 3 shows considerable variation in the estimated effect sizes and their associated confidence limits. However, the figure displays the classic meta-analysis pattern, with the majority (13 of the 19 studies) showing positive estimates and the remainder (6 studies) showing estimates that were negative or essentially zero. The cvb study (row 1), the cph study (row 2), and the cpp studies (rows 3 to 12) tended to provide the tightest signal, with generally narrower CIs and positive estimates. For 4 of the 19 studies (cph, cppbtn, cpprmd, cppbtm), the CIs excluded zero. None of the studies with negative estimates had CIs that exclude zero. Indeed, the negative studies tended to have generally wider CIs. The 2 smallest studies (cvb and pbt), which each had only around 150 children, had 1 study showing a positive effect and 1 a negative effect.

The visual impression from the forest plot is consistent with the technical finding, namely, a modest but significant positive association between growth velocity and child cognition. The measure of between-study variance, ${\text{τ}}^{2}$, estimated using restricted maximum likelihood, was ${\text{τ}}^{2}$ (SE) = 0.03 (0.046). The $I^{2}$ statistic,^45^ which indicates the percentage of total variation across studies that are due to heterogeneity rather than chance, is estimated to be 22%. As expected, results based on fitting a linear mixed-effects model to all the combined data were essentially identical. In terms of potential sources of heterogeneity, there are a number of possibilities. For example, there may be regional and country-specific variation in terms of health care practices or interventions that may be implemented in settings where a child is showing poor growth. Variation in nutritional practices may also have an influence, and this factor was not assessed. Finally, there may be cultural factors that influence the suitability of various standardized tests in measuring child cognition.

## Discussion

We have demonstrated the use of propensity scoring to perform meta-analysis in settings where covariates vary between studies. By calculating propensity scores separately for each study and then using the propensity score as a covariate in our regression model, we can conduct meta-analysis using a common model across our studies, as opposed to performing meta-analysis on measures that come from different models. Our method is applicable to distributed data systems where the individual shards of data may not have the same structure. In these cases, the use of propensity scores firstly unifies the model that the nodes of the distributed data system will fit. In addition, using meta-analysis allows us to combine the analysis performed using individual shards. Furthermore, our method can be easily implemented in distributed computing paradigms such as MapReduce, and Divide and Recombine, where the Map and Divide stages correspond to the regression using propensity scores, and the Reduce/Recombine steps correspond to the random-effects meta-analysis.^46^

Our motivating example used meta-analysis to assess the relation between physical growth rate in the first year of life and cognition measured later during childhood. There were several challenges with the analysis because the studies being combined varied in terms of the available covariates, timing of various growth measures, and precise nature of the measured outcomes. Some children were also missing some of the covariate measures. We used the HAZ as the physical measure of growth, although depending on the goal of the analysis, subject matter experts may prefer other physical growth measures, such as WAZ or head circumference size. To model the first-year physical growth rate, we used a broken-stick approach, which worked well and allowed for the possibility that the studies varied in the timing at which growth measures were taken.^41^

Our focus was to describe the use of propensity score methodology, combined with multiple imputation, to address the fact that the studies varied in terms of the precise nature of measured covariates. We employed multiple imputation to handle missing covariates to avoid potential biases in estimating the propensity score. Following the multiple imputation process, we estimated a propensity score using the generalized propensity score approach. We fit a common outcome model across all studies by including the estimated propensity scores as an additional covariate in our outcome regression model. In this work, we assumed that a linear propensity model is correct. Although there is a possibility of model misspecification, works such as that by Drake^47^ has shown that more bias to the estimates is introduced when the outcome or response model is misspecified than when the propensity model is misspecified.^47^

We outlined a workflow that can be followed by practitioners who conduct IPD meta-analysis in the presence of heterogeneous and partially observed covariates. Specifically, we imputed the missing values within each study and estimated a propensity score for each imputed dataset. We then followed the “RR then MA” approach (as illustrated in Figure 2) to conduct a two-stage IPD meta-analysis and obtain an estimated effects sizes across all studies. We then showed how to conduct one-stage IPD meta-analysis in the presence of heterogeneous and partially observed covariates. The approach we described for the one-stage IPD meta-analysis is slightly different from the approach for the two-stage IPD meta-analysis. Specifically, first, one needs to group the imputed datasets for each study according to the imputation order and meta-analyze these combined imputed datasets to obtain effect size. Then, the process must be repeated through all the combined datasets and Rubin’s rules applied to pool the effect sizes obtained from each combined imputed dataset. In one-stage analysis, we first fit a hierarchical model and then applied to Rubin’s rules to combine resulting estimates. For this reason, the heterogeneity of the meta-analysis results may include both the heterogeneity between studies and the heterogeneity introduced due to the imputation process.

In both approaches in this paper, we imputed missing covariates within each study. With individual subject data meta-analysis, a more complex approach suggested by Quartagno and Carpenter^7^ is to use multilevel joint modeling with a random study-specific covariance matrix. If we use this imputation method, we must treat all the data as a single dataset. Therefore, when we apply imputation, we perform meta-analysis first and then apply Rubin’s rules. With this setup, after imputation, we can fit our random-effects meta-analysis model for each imputed dataset. We then combined the estimates from the meta-analysis models using Rubin’s rules. This is equivalent to performing regression on each of the imputed data and then pooling them.

## Conclusion

We have shown that the use of propensity scores provides an attractive and flexible option for conducting meta-analysis in settings where the studies being combined have collected data on different sets of covariates. Missing data issues can be easily addressed using multiple imputation. With this methodology, researchers can perform an IPD meta-analysis using a common model across all studies, which is particularly important when conducting the one-stage meta-analysis. An important caveat is that each of the studies included in the meta-analysis should be well designed and reliable in terms of providing a good estimate of the overall effect of interest. This includes, among other things, that adequate covariates have been measured within each individual study to remove confounding effects. Ensuring that this is the case is not unique to our approach; instead, it is an essential step in any meta-analysis. In settings where there are concerns regarding whether individual studies have measured adequate covariates, consideration should be given to omitting these studies from the meta-analysis. However, our simulation analysis suggests that as long as most of the studies meet this criterion, the overall meta estimates may only be slightly biased. A potential limitation of the approach is that it makes several fairly strong modeling assumptions. For example, the framework assumes a linear relationship between the exposure and the outcome of interest, after adjusting for covariates.

In practice, it will be important to assess model goodness of fit and to consider alternatives such as taking logs or other transformations of the exposure variable. Consideration can also be given to potential interactions between the exposure variable and the propensity scores. However, the appeal of the proposed framework is that by incorporating study-specific covariates via propensity scores, standard practice for model assessment and goodness of fit can then apply.

## Supplementary Material

Supplemental Digital Content

## Figures and Tables

**Table 1:** Cognitive outcome measure, mean age (in years) of subjects at measurement of cognitive outcomes, number of subjects, and observed covariates for each study used in the simulation

Study	Cognitive outcome measure	Number of subjects	Age	Measured covariates
Consortium of Health-Orientated Research in Transitioning Societies (COHORTS)-Philippines^31^ (cph)	General IQ	2252	11	Gravidity, parity, sex, socioeconomic covariates, access to health care, caregiver’s education
Collaborative Perinatal Project^30^ (cpp)	Weschler Intelligence Scale for Children (2 sites used the Stanford Binet test)	38,730	7	Gravidity, parity, sex, socioeconomic covariates, height, number of prenatal visit, APGAR scores 1 site also collected data on gestational age
CMC Vellore Birth Cohort 2002^32^ (cvb)	General IQ	147	8	Gravidity, parity, sex, socioeconomic covariates, medical history, details about the house that the subject lives in
Growing Up in Singapore Towards healthy Outcomes^38^ (gto)	Bayley Scales of Infant Development	151	2	Gestational age at birth, gravidity, parity, sex, socioeconomic covariates, height, father’s age, APGAR scores, mode of child delivery, breastfeeding duration, prenatal alcohol consumption
JiVitA-3: Impact of antenatal multiple micronutrient supplementation on infant mortality^33^ (jta)	Bayley Scales of Infant Development	677	2	Gestational age at birth, gravidity, parity, sex, socioeconomic covariates, mother’s weight, maternal age, vaccination information, living conditions
MRC Keneba^37^ (nhb)	Wechsler Abbreviated Scale of Intelligence	398	2	Gestational age at birth, gravidity, parity, sex, socioeconomic covariates, living conditions, number of persons in house
Peru Persistent Diarrhea study^35^ (pvd)	General IQ	421	2	Gestational age at birth, gravidity, parity, sex, socioeconomic covariates, breastfeeding duration
Promotion of Breast Feeding Interventional Trial^34^ (pbt)	Wechsler Abbreviated Scale Intelligence (WASI)	147	6	Gestational age at birth, gravidity, parity, sex, socioeconomic covariates, stratum of geographical area, breastfeeding duration, maternal heigh, maternal weight, smoking during pregnancy, prenatal alcohol exposure, pregnancy complications
Social Medical Survey of Children attending Child health Clinics^36^ (scc)	General IQ	397	6	Gestational age at birth, gravidity, parity, sex, socioeconomic covariates, APGAR scores, father’s demographic information, smoking during pregnancy

## Appendix I Observed covariates by study

**Table:**

Study name	Observed covariates
cvb	Sex Mode of child delivery Child cried at birth Mother years of education in pre-defined years Social-economic status of parent in defined categories Maternal num of abortions Maternal num of still births Maternal num of living children Location of delivery Highest education of caregiver in defined number of years Highest education of caregiver in family defined number of years Highest education head of household in family defined number of years Number of adults in the house Number rooms in house Has car Clothing cabinet in home Type of cooking fuel as pre-defined types Place for cooking as pre-defined places Family type as pre-defined types Occupation (primary) no defined types Parent own home Type of roof over home Tape recorder Percent of days with diarrhea
cph	Sex Gestational age at birth (days) Breastfeeding duration Ratio of all children to adults Child dependency ratio Crowding index Water availability at water source Access to health care Use of preventive health services Total family income Number of adult females Number of adult males Maternal age at birth of child (years) Maternal height (cm) Mothers marital status (num) Mother years of education Father years of education Social economic status Maternal age at birth of first child (years) Maternal parity Maternal num of female children Maternal num of male children
cppbtm	Sex Birth weight (g) APGAR Score 1 min after birth APGAR Score 5 mins after birth Maternal age at birth of child (years) Mother’s race Maternal height (cm) Mother’s marital status Mother years of education Father years of education Maternal parity Maternal num of abortions Maternal num of still births Number of antenatal health care visits
cppbtn	Sex Gestational age at birth (days) Birth weight (g) APGAR Score 1 min after birth APGAR Score 5 mins after birth Maternal age at birth of child (years) Mother’s race Maternal height (cm) Mother’s marital status Mother years of education Father years of education Maternal parity Maternal num of abortions Maternal num of still births Number of antenatal health care visits
cppbfl	Sex Birth weight (g) APGAR Score 1 min after birth APGAR Score 5 mins after birth Maternal age at birth of child (yrs) Mother’s race Maternal height (cm) Mother’s marital status Mother years of education Father years of education Maternal parity Maternal num of abortions Maternal num of still births Number of antenatal health care visits
cppmph	Sex Birth weight (g) APGAR Score 1 min after birth APGAR Score 5 mins after birth Maternal age at birth of child (years) Mother’s race Maternal height (cm) Mother’s marital status Mother years of education Father years of education Maternal parity Maternal num of abortions Maternal num of still births Number of antenatal health care visits
cppmnp	Sex Birth weight (g) APGAR Score 1 min after birth APGAR Score 5 mins after birth Maternal age at birth of child (years) Mother’s race Maternal height (cm) Mother’s marital status Mother years of education Father years of education Maternal parity Maternal num of abortions Maternal num of still births Number of antenatal health care visits
cppnwo	Sex Gestational age at birth (days) Birth weight (g) APGAR Score 1 min after birth APGAR Score 5 mins after birth Maternal age at birth of child (years) Maternal height (cm) Mother’s marital status Mother years of education Father years of education Maternal parity Maternal num of abortions Maternal num of still births Number of antenatal health care visits
cppnwk	Sex Birth weight (g) APGAR Score 1 min after birth APGAR Score 5 mins after birth Maternal age at birth of child (years) Mother’s race Maternal height (cm) Mother’s marital status Mother years of education Father years of education Maternal parity Maternal num of abortions Maternal num of still births Number of antenatal health care visits
cppphl	Sex Gestational age at birth (days) Birth weight (g) APGAR Score 1 min after birth APGAR Score 5 mins after birth Maternal age at birth of child (years) Mother’s race Maternal height (cm) Mother’s marital status Mother years of education Father years of education Maternal parity Maternal num of abortions Maternal num of still births Number of antenatal health care visits
cpppld	Sex Birth weight (g) APGAR Score 1 min after birth APGAR Score 5 mins after birth Maternal age at birth of child (yrs) Mother’s race Maternal height (cm) Mother’s marital status Mother years of education Father years of education Maternal parity Maternal num of abortions Maternal num of still births Number of antenatal health care visits
cpppvd	Sex Birth weight (g) APGAR Score 1 min after birth APGAR Score 5 mins after birth Maternal age at birth of child (yrs) Mother’s race Maternal height (cm) Mother’s marital status Mother years of education Father years of education Maternal parity Maternal num of abortions Maternal num of still births Number of antenatal health care visits
cpprmd	Sex Birth weight (g) APGAR Score 1 min after birth APGAR Score 5 mins after birth Maternal age at birth of child (years) Mother’s race Maternal height (cm) Mother’s marital status Mother years of education Father years of education Maternal parity Maternal num of abortions Maternal num of still births Number of antenatal health care visits
gto	Sex Gestational age at birth (days) Breastfeeding duration (days) Age of menarche Mode of child delivery APGAR Score 1 min after birth APGAR Score 5 mins after birth Maternal age at birth of child (years) Mother ethnicity Maternal height (cm) Mother’s marital status Mother’s work Father’s age at birth of child (years) Estimated blood loss mL Amount of beer during pregnancy as pre-defined level Maternal parity Maternal num pregnancies Type of floor in house Total family income in as pre-defined levels
jta	Sex Gestational age at birth (days) Child received BCG vaccine Child received OPV vaccine Child of multiple births Mode of child delivery Child cried after birth Maternal age at birth of child (years) Maternal weight kg Mother years of education Father smoking status Father years of education Father type of work as pre-defined levels Maternal age in years at time of first delivery Maternal parity Living standard index Number of adults in house Number of children less than five in house Productive asset index Source of bathing water Source of cooking water Source of washing kitchen utensils Source of drinking water Source of sanitation water
nhb	Sex Percent of days with diarrhea Maternal age at birth of child (years) Mother years of education Bench in the home Bicycle Chair in the home Clothing cabinet in home Cot in the home Electricity Fan in the home Type floor in house Stored food is covered Household food deficit Stored drinking water is covered Frequency of water retrieval in 24 hrs Water source Source of cooking water Source of washing kitchen utensils Source water other use Source drinking water Source for handwashing for bottle clean Source for handwashing for defecation Source for handwashing for eating Source for handwashing for feeding child Drinking stored water Total family income Taka per month Motorcycle Number of persons in house Has natural gas in home Number of windows in home Phone Radio Type of roof over home Type of sanitary facility Sewing machine Table in the home Television Type walls in house Wash hands cleaning child’s bottle Wash hands after defecation Wash hands prior to eating Wash hands prior to nursing Watch in the home
pbt	Sex Gestational age at birth in days Stratum geographical area Feeding practice pre-defined group Breastfeeding duration in days Mode of child delivery APGAR Score 1 min after birth APGAR Score 5 mins after birth Maternal age at birth of child in years Maternal height in cm Maternal weight in kg Maternal BMI in kg/m^2^ Mother’s marital status Mother years of education Mother type or work Father’s age at birth of child in years Father’s weight in kg Father years of education Father type of work Mom smoked during pregnancy Amount of alcohol in pre-defined steps Maternal num of living children Pregnancy complications or risk factors
pvd	Sex Feeding practice Breastfeeding duration in days Maternal age in years at time of first delivery Maternal parity Maternal num of still births Maternal num of live births Maternal num of living children Location of delivery Child received BCG vaccine
scc	Sex Feeding practice Gestational age at birth in days Mode of child delivery APGAR Score 1 min after birth APGAR Score 5 mins after birth Maternal age at birth of child in years Maternal height in cm Mother years of education Father’s age at birth of child in years Father’s height in cm Father years of education Num cigarettes mom smoked per day NA Maternal num pregnancies Maternal num of still births Maternal num of living children Maternal num of deceased children Location of delivery Person conducting the delivery
