# Supplementary material for: Meta-analysis on studies with heterogeneous and partially observed covariates
Source: JBI Evid Synth. Author manuscript; Available in PMC 2024 Jun 26. (PMC11200172; doi:10.11124/JBIES-23-00078)
Supplement: Supplemental Digital Content [file NIHMS1968758-supplement-Supplemental_Digital_Content.pdf]

JBI EVIDENCE SYNTHESIS

Supplemental content for "Meta-analysis on studies with heterogeneous and partially observed covariates"

Simulation setting

We consider K studies with 1 continuous outcome and an exposure variable for each study. For each study, we generated 3 covariates, X_1, X_2, X_3, in such a way that they were all intercorrelated with each other and that they were true confounders. This means that the 3 covariates were correlated with the exposure variable and with the outcome, even after accounting for the exposure. We generated the interrelationships between all these variables in such a way that the precise relationships varied from study to study. Below is a more detailed description of how our simulation was conducted.

We first generated $X_{1jk} \sim N(0,1)$. We then we generated X_{2jk} to depend on X_{1jk} based on the following linear regression model:

$$X_{2jk} = \beta_k X_{1jk} + e_{jk}^{x}$$

where $e_{jk}^{x} \sim N\left(0, \sigma_k^2\right)$. To allow for study-to-study variability, study-specific values of β_k were chosen by generating 10 random values from $N\left(\mu_\beta, \sigma_\beta^2\right)$ (see Table S1 for values). Specific values of σ_k^2 were chosen by generating 10 random values from $N\left(\mu_{\sigma_k^2}, \sigma_{\sigma_k^2}^2\right)$. Next, we generated X_{3jk} to depend on X_{1jk} and X_{2jk} using the following linear regression model:

$$X_{3jk} = \alpha_{1k} X_{1jk} + \alpha_{2k} X_{2jk} + r_{jk},$$

where $r_{jk} \sim N\left(0, \gamma_k^2\right)$. Again, study-specific values of α_{1k}, α_{2k}, and γ_k^2 were generated from normal distributions.

We then generated the exposure variable E, conditional on the 3 covariates,

$$E_{jk} = \theta_{1k} X_{1jk} + \theta_{2k} X_{2jk} + \theta_{3k} X_{3jk} + \epsilon_{jk},$$

Akkaya Hocagil T, Hwang H, Jacobson JL, Jacobson SW, Ryan LM. Meta-analysis on studies with heterogeneous and partially observed covariates. JBI Evid Synth. 2024;22(3). https://doi.org/11.11124/JBIES-23-00078.

where $\epsilon \sim N(0, \eta_k^2)$, again with the various coefficients generated from common distributions to induce study-to-study variability. Finally, we generated the outcome from the following linear regression model:

$$Y_{jk} = \delta_{0k} + \delta_{1k}E_{jk} + \delta_{2k}X_{1jk} + \delta_{3k}X_{2jk} + \delta_{4jk}X_{3jk} + e_{jk},$$

where Y_{jk} would be the random variable representing response for study k and individual j. We let δ_{1k} vary about some average exposure effect across studies, with $\hat{\delta}_{1k} \sim N(\delta_{1k}, \tau^2)$. To allow for study-to-study variability, we generated the true effect size for the exposure δ_{1k} from a normal distribution with mean 5 and variance 0.8. The parameters δ_{2k}, δ_{3k}, and δ_{4k} characterize the effects of the X_1, X_2, and X_3, respectively, for a given level of exposure. We also assume $e_k \sim N(0, \zeta_k^2)$. Specific values of all simulation parameters are provided in Table S1.

We assumed a balanced design, with a sample size of 200 for each study. For each dataset, we performed 2 types of meta-analysis (ie, two-stage meta-analysis while controlling for confounders via the generalized propensity score approach and one-stage individual participant data meta-analysis while controlling for confounders via propensity score. We considered 2 specific scenarios: we considered a setting where the data on all covariates are observed for all the studies (Scenario A), and a setting where the data on covariate X_2 was not collected for study 2 and the data on covariate X_3 was not collected for study 6 (Scenario B). Under Scenario B, we considered 3 types of meta-analysis: two-stage meta-analysis using a generalized propensity score based on all the available covariates in each study, one-stage IPD meta-analysis using a generalized propensity score based on all the available covariates in each study, and finally a one-stage individual participant data meta-analysis based on a generalized propensity score that uses only the completely observed covariates, (ie, X_1).

Akkaya Hocagil T, Hwang H, Jacobson JL, Jacobson SW, Ryan LM. Meta-analysis on studies with heterogeneous and partially observed covariates. JBI Evid Synth. 2024;22(3). https://doi.org/11.11124/JBIES-23-00078.

Table S1: Simulation parameters

Parameter	Mean	SD
σ_k^2	1.5	0.20
β	13.1	23.5
α_1	3.7	1.2
α_2	2.1	0.7
γ_k^2	2.5	0.8
θ_1	0.9	0.4
θ_2	1.7	1.3
θ_3	1.5	1
η_k^2	2.2	0.8
δ_1	5.0	0.8
δ_2	1.3	0.7
δ_3	2.2	1.1
δ_4	2.4	1.4
ζ_k^2	2.3	0.9

We evaluated the performance of our approach in the simulation setting described over 1000 iterations. The estimate of interest was the average exposure effect. To allow for a comprehensive comparison, performance was assessed on a range of metrics: empirical bias, average model-based standard error, empirical standard error, and coverage probability.[1] A brief definition of each metric is provided below, and results are summarized in Table S2:

- Empirical bias: Empirical bias is frequently of central interest and quantifies whether a method targets θ on average.
- Average model-based standard error: Model-based standard errors that are estimated from the fitted model for each simulated dataset.
- Empirical standard error: The empirical standard error is a measure of the precision or efficiency of the estimator of θ. It depends only on θ_i and does not require knowledge of θ. The empirical standard error estimates the long-run standard deviation of θ_i over the number of simulated datasets.
- Coverage probability: Coverage of CIs is a key property for the long-run frequentist behavior of an estimator. It is defined as the probability that a CI contains θ.

Table S2: Results of the simulation studies

		EBIAS	ASE	ESE	CP
All relevant covariates observed	Two-stage meta-analysis	–0.005	0.285	0.289	0.93
	One-stage IPD meta-analysis	–0.006	0.268	0.289	0.99
Two studies have a missing covariate	Two-stage meta-analysis	0.189	0.319	0.288	0.92
	One-stage IPD meta-analysis	0.196	0.301	0.289	0.90
Fully observed covariate only	One-stage IPD meta-analysis	1.766	0.483	0.287	0.007

ASE, average model-based standard error; CP, coverage probability; EBIAS, empirical bias; ESE, empirical standard error; IPD, individual participant data

SAS and R code to fit the models described in the "Application to child growth data" section

In SAS, the PROC MIXED procedure can be used. For example, the relevant code would be:

```
proc mixed data= method=;
class STUDY;
model Y = Z S*STUDY/ SOLUTION;
random Z /SOLUTION ; repeated /Group=STUDY;
run;
```

First we need to specify input data set and the estimation method. The CLASS statement names the classification variables to be used in the model. In our model, the classification variable is the study ID. The MODEL statement names a single dependent variable and the fixed effects, which determine the matrix of the mixed model, and SOLUTION requests that a solution for the fixed-effects parameters be produced. The RANDOM statement defines the random effects in the mixed model, and SOLUTION displays solutions of the estimated random effect.

To do the analysis in R, it is necessary to install and load the R package lme4 to fit a random-effects model in R. *lmer* is the function that fits the random effects model:

```
Install.packages("lme4")
library(lme4)
Model  <- lmer(Y ~0+STUDY+ Z + S:STUDY+
         (0 + Z | STUDY),
       data =)
```

JBI EVIDENCE SYNTHESIS

In this code, Y is the outcome, Z is the exposure variable, and S:STUDY is the interaction term for the study (STUDY) and the estimated propensity score (S). It is important to include this interaction to account for the fact propensity scores are estimated within each study. (0 + Z | STUDY) specifies the random effect (Z) and class variable (STUDY).

1. Morris TP, White IR, Crowther MJ. Using simulation studies to evaluate statistical methods. Stat Med. 2019;38(11):2074–102.
